# Silencing of RpATG6 impaired the yolk accumulation and the biogenesis of the yolk organelles in the insect vector *R*. *prolixus*

**DOI:** 10.1371/journal.pntd.0006507

**Published:** 2018-05-16

**Authors:** Priscila H. Vieira, Larissa Bomfim, Georgia C. Atella, Hatisaburo Masuda, Isabela Ramos

**Affiliations:** 1 Laboratório de Bioquímica de Insetos, Instituto de Bioquímica Médica, Universidade Federal do Rio de Janeiro, Rio de Janeiro, Brazil; 2 Laboratório de Bioquímica de lipídeos e lipoproteínas, Instituto de Bioquímica Médica, Universidade Federal do Rio de Janeiro, Rio de Janeiro, Brazil; Yale School of Public Health, UNITED STATES

## Abstract

In oviparous animals, the egg yolk is synthesized by the mother in a major metabolic challenge, where the different yolk components are secreted to the hemolymph and delivered to the oocytes mostly by endocytosis. The yolk macromolecules are then stored in a wide range of endocytic-originated vesicles which are collectively referred to as yolk organelles and occupy most of the mature oocytes cytoplasm. After fertilization, the contents of these organelles are degraded in a regulated manner to supply the embryo cells with fundamental molecules for *de novo* synthesis. Yolk accumulation and its regulated degradation are therefore crucial for successful development, however, most of the molecular mechanisms involved in the biogenesis, sorting and degradation of targeted yolk organelles are still poorly understood. ATG6 is part of two PI3P-kinase complexes that can regulate the recruitment of the endocytic or the autophagy machineries. Here, we investigate the role of RpATG6 in the endocytosis of the yolk macromolecules and in the biogenesis of the yolk organelles in the insect vector *Rhodnius prolixus*. We found that vitellogenic females express high levels of RpATG6 in the ovaries, when compared to the levels detected in the midgut and fat body. RNAi silencing of RpATG6 resulted in yolk proteins accumulated in the vitellogenic hemolymph, as a consequence of poor uptake by the oocytes. Accordingly, the silenced oocytes are unviable, white (contrasting to the control pink oocytes), smaller (62% of the control oocyte volume) and accumulate only 40% of the yolk proteins, 80% of the TAG and 50% of the polymer polyphosphate quantified in control oocytes. The cortex of silenced oocytes present atypical smaller vesicles indicating that the yolk organelles were not properly formed and/or sorted, which was supported by the lack of endocytic vesicles near the plasma membrane of silenced oocytes as seen by TEM. Altogether, we found that RpATG6 is central for the mechanisms of yolk accumulation, emerging as an important target for further investigations on oogenesis and, therefore, reproduction of this vector.

## Author summary

In oviparous animals all the nutrients and energy needed for the embryo cells are previously stored in the eggs, in the form of what we call yolk. The yolk is the food of the embryo, and, like our food, it needs to be broken down into nutrients and energy to supply the embryo cells rapid growth and division. The yolk-macromolecules are maternally synthesized, accumulated in the oocytes into specific organelles and, after fertilization, they are degraded in a regulated manner throughout early embryogenesis. The cellular and molecular mechanisms that govern these components accumulation and degradation are still mostly unknown. This is our interest in this work. We found that one specific gene (RpATG6) is crucial for the endocytosis of the yolk components by the oocytes in the insect vector of Chagas disease *R*. *prolixus*. Insects silenced for this gene generate oocytes lacking most of their yolk components, and, as a consequence, their F1 embryos are not viable. Thus, these findings are important in the context of vector population control and can help us to further understand the molecular mechanisms of yolk uptake and oocyte formation in these animals.

## Introduction

Oocytes are remarkable cells in the matter of accumulating macronutrients. Oviparous animals have evolved to produce germline cells that not only enter meiosis to generate a gamete, but also differentiate into a giant cell designed to support embryo growth. Once fertilized, the egg is able to fulfill embryo development away from the maternal body. To accomplish that, an oocyte typically grows up to 5,000x its original size by accumulating macromolecules in a highly specialized cytoplasm with maternal mRNAs, proteins, ribosomes, mitochondria and so on. That stock of macronutrients is referred to as yolk. The yolk components are entirely made by the mother in a major metabolic challenge where proteins, lipids and carbohydrates are synthesized and delivered to the oocytes mostly by endocytosis. The yolk macromolecules are stored in a wide range of endocytic-originated vesicles which are collectively referred to as yolk organelles and occupy more than 99% of the mature oocytes cytoplasm [1–3]. After fertilization, the programmed degradation of the contents stored in the yolk organelles is crucial to support the anabolic metabolism of the growing embryo and, therefore, successful development.

ATG6/beclin1 is a multifunctional protein that appears to participate in several functions including endocytosis [4, 5], autophagy [6–10], aging [11], immunity [12], cell death [13–16] and others. ATG6 contains a BCL-2 homology domain (BH3), a coiled-coil domain (CCD) and an evolutionarily conserved domain (ECD), which together enable multiple interactions with different target proteins. Through ECD, two distinct PI3K complexes can be formed, the complex I binds ATG14 and initiates the autophagosome formation by accumulation of PI3P, and the complex III that binds VPS15, a vacuolar protein sorting protein, which also results in the accumulation of PI3P but now recruiting the endocytic machinery [17]. Genetic loss-of-function studies have revealed that ATG6 orthologues can participate in endocytosis and autophagy in different organisms including *A*. *thaliana*, *D*. *melanogaster*, *C*. *elegans* and mice [5, 18–20], but the molecular mechanisms that regulate the formation of the two distinct PI3K complexes are still poorly understood.

Because the oocyte is an endocytosis-specialized cell, we investigate here the role of ATG6 in the uptake of the yolk components using the oocytes of the insect *Rhodnius prolixus* as a model. *R*. *prolixus* is an insect vector of Chagas disease, which is one of the neglected tropical diseases that are important in Latin America and Africa (WHO, 2017). Currently 8 million people are estimated to be infected by Chagas disease, and vector control is still the most useful method to prevent this illness [21]. As embryo development relies entirely on the yolk nutritional reserves, mobilization of the yolk components is crucial for embryo viability, and we hope that molecular investigations on its mechanisms may reveal targets for interference in vector’s reproduction and eventually contribute to the elaboration of new strategies for population control. *Rhodnius* genome was recently published and transcriptome databases are available at Vector Base [22]. Parental gene silencing can be consistently performed via RNAi [23, 24] and, since it is a strictly hematophagous insect, oogenesis is highly synchronized with the blood meal, which is convenient if one aims to monitor oocyte development.

We silenced the one isoform of ATG6 found in the genome of *R*. *prolixus (RpATG6)* and found that it results in the accumulation of yolk proteins in the insect hemolymph during vitellogenesis as a consequence of poor uptake by the oocytes. The mature oocytes from silenced females are not able to properly form the endocytic-originated yolk organelles in the cortex, and, as a result, they are smaller and embryos are unviable. The amounts of the main yolk proteins, vitellin and RHBP, and the non-protein yolk components, TAG and polyphosphate (PolyP), are severely compromised. The importance of ATG6 in the yolk organelles biogenesis and proper oocyte formation is discussed.

## Materials and methods

### Bioinformatics

The sequence of RpATG6 was obtained from the *R*. *prolixus* genome and transcriptome databases (Rpro C3.2) from Vector Base (www.vectorbase.org). The sequence of RpATG6 was identified by a local BLAST database made on Bio Edit software. The Drosophila ATG6 orthologue (AAF56227.1) was used as the first template for identification. Values of similarity and identity were predicted using SIA software and conserved domains were predicted using PFAM.

### Insects

Insects were maintained at a 28 ± 2°C controlled temperature and relative humidity of 70–80%. The experimental animals used were adult females directly fed in live-rabbit blood at 21 days intervals. All animal care and experimental protocols were approved by guidelines of the institutional care and use committee (Committee for Evaluation of Animal Use for Research from the Federal University of Rio de Janeiro, CEUA-UFRJ #01200.001568/2013-87, order number 155/13), under the regulation of the national council of animal experimentation control (CONCEA). Technicians dedicated to the animal facility conducted all aspects related to animal care under strict guidelines to ensure careful and consistent animal handling.

### Extraction of RNA and cDNA synthesis

All organs were dissected 6 days after blood meal and homogenized in Trizol reagent (Invitrogen) for total RNA extraction. Reverse transcription reaction was carried out using the High Capacity cDNA Reverse Transcription Kit (Applied Biosystems), using 1 μg of total RNA after RNase-free DNase I (Invitrogen) treatment, all according to the manufacture’s protocol.

### PCR/qPCR

Specific primers for RpAtg6 sequence were designed to amplify a 226 bp fragment in a PCR using the following cycling parameters: 10 min at 95°C, followed by 35 cycles of 15 s at 95°C, 45 s at 52°C and 30 s at 72°C and a final extension of 15 min at 72°C. Amplifications were observed in 2% agarose gels. Quantitative PCR (qPCR) was performed in a StepOne Real-Time PCR System (Applied Biosystems) using SYBR Green PCR Master Mix (Applied Biosystems) under the following conditions: 10 min at 95°C, followed by 40 cycles of 15 s at 95°C and 45 s at 60°C. qPCR amplification was performed using the specific primers 5’CCGCTCCTGTAGACTGGTC3’(F), 3’GCCACCATCGCAGCATCAAATTTTG5’ (R). Rp18S was used as endogenous control, with the following primers: 5’TCGGCCAACAAAAGTACACA3’(F), 3’TGTCGGTGTAACTGGCATGT5’(R). The relative expression and ΔC_t_ values were calculated from obtained C_t_ (cycle threshold) values [25].

### RNAi silencing

dsRNA was synthesized by MEGAScript RNAi Kit (Ambion Inc) using primers for *RpATG6* specific gene amplification with the T7 promoter sequence 5’GCAGTTTGGGAGAACATACTCTCG3’(F); 3’CTGTACACTTCTGTGTTCATCTTCC5’(R) designed to target a region of 595bp. Unfed adult females were injected with 1 μg dsRNA [26] and fed 2 days later. Knockdown efficiency was confirmed by PCR and qPCR at different days after blood meal. The bacterial *MalE* gene was used as a control dsRNA [27]. Adult females injected with dsRNA were fed and transferred to individual vials. The mortality rates and the number of eggs laid were recorded daily and weekly, respectively. Additional measurements are described below.

### PI3P detection by thin-layer chromatography (TLC) and quantification

Silenced and control females were fed with blood enriched with 32Pi [28] using a special feeder [29]. On the tenth day after a blood meal, the chorionated oocytes were collected and subjected to lipid extraction [30]. The lipid extracts were chromatographed on oxalate-impregnated, thin-layer silica plates (G-60; 0–25 mm thickness) using chloroform–acetone–methanol–acetic acid–water (40: 15:13:12:8, by vol.) developing solvent [31]. The radioactivity was analyzed in a laser scanner Cyclone® Plus Storage Phosphor System (Perkin Elmer). To visualize the lipids, the plates were immersed for 10 s in a charring solution consisting of 3% CuSO_4_ and 8% H_3_PO_4_ (v/v) and heated to 110°C for 10 min. The charred TLC plates were then subjected to densitometric analysis using Adobe Photoshop CC 2015 software. The phospholipids spots were identified by comparison with standards run in parallel.

### Egg homogenates and SDS-PAGE

Control and silenced eggs were collected at 24h of embryogenesis. Pools of 4 eggs were homogenized in 100 μl of phosphate buffered saline (PBS) containing a cocktail of protease inhibitors (Aprotinin 0.3 μM, leupeptin 1 μg/μl, pepstatin 1 μg/μl, PMSF 100 μM and EDTA 1 mM). 30 μg of total protein were loaded in each lane of a 13% SDS-PAGE. Gels were stained with silver nitrate [32].

### Hemolymph extraction and SDS-PAGE

Hemolymphs of silenced and control females were collected at 7 days after blood feeding from a pool of 3 insects. Once collected, the hemolymph was diluted 2x in PBS containing protease inhibitors (aprotinin 0.3 μM, leupeptin 1 μg/μl, pepstatin 1 μg/μl, PMSF 100 μM and EDTA 1 mM), and approximately 8 mg of phenylthiourea. 2 μl of hemolymph was loaded in each lane of a 13% SDS-PAGE, corresponding to 30–40 μg of protein for dsMal samples and 60–70 μg for dsRpATG6 samples.

### Determination of protein content

The total amount of protein in the silenced and control eggs and hemolymphs was measured by the Lowry (Folin) method, using as standard control 1–5 μg of BSA [33] in a E-MAX PLUS microplate reader (Molecular devices) using SoftMax Pro 5.0 as software.

### Determination of glycogen content

Glycogen content was determined in 100 μl of the egg homogenate described above using the Glucox 500 kit (Doles reagents) following the manufacture’s protocol. The samples were incubated for 4h at 40 C° in the presence of 20 μl (1U) of amyloglicosidase. Controls were prepared under the same conditions, but in the absence of enzyme.

### Determination of triacylglycerol (TAG) content

Eggs homogenates were prepared, as described above, and were used to determine the total amount of neutral lipids such as TAG. Lipid extraction was performed for 2 h in a tube containing 5 mL chloroform-methanol-water solution (2:1:0.8, v/v), with intermittent shaking. The mixture was centrifuged at 1500 *g* for 30 min at 4°C, the supernatant was collected and the pellet subjected to a second lipid extraction (1 h). To the pooled supernatants, 5 mL water and 5 mL chloroform were added, the mixture was shaken and, after centrifugation, the organic phase was removed and dried under nitrogen. Extracted lipids corresponding to 1 egg were analyzed by one-dimensional thin-layer chromatography (TLC) for neutral lipids [34]. To visualize the lipids, the plates were immersed for 10 s in a charring solution consisting of 3% CuSO_4_ and 8% H_3_PO_4_ (v/v) and heated to 110°C for 10 min. The charred TLC plates were then subjected to densitometric analysis using Adobe Photoshop CC 2015 software. The endogenous TAG spots were identified by comparison with TAG standards run in parallel.

### Determination of PolyP content

Quantification of PolyP in egg homogenates was done using a general protocol based in the fluorimetric analysis of the characteristic emission of the DAPI-PolyP complex, as described before by [35]. Excitation wavelength was 420 nm and the emission wavelength was of 535 nm. PolyP_65_ was used for a standard curve ranging from 90 to 540 ng of PolyP. Measurements were made using a 2030 Victor X5 fluorometer (Perkin Elmer).

### Light microscopy

Opercula of the eggs were carefully detached using a sharp razor blade under the stereomicroscope. Vitellogenic oocytes and opercula-free eggs were fixed by immersion in 4% freshly prepared formaldehyde in 0.1 M cacodylate buffer, pH 7.2 for 12 h at room temperature. Samples were washed 3 times for 10 minutes in the same buffer and embedded in increasing concentrations (25%, 50%, 75% and 100%) of OCT compound medium (Tissue-TEK) plus 20% glucose as a cryoprotectant, for 12 h for each of the concentrations. Once infiltrated in pure OCT, 14 μm transversal sections of the oocytes and eggs were obtained in a cryostat. The slides were mounted in glycerol 50% followed by observation in a Zeiss Observer.Z1 equipped a Zeiss Axio Cam MrM operated in a differential interferencial contrast (DIC) mode.

### Electron microscopy

Vitellogenic oocytes were fixed for 4–6 hours in 2.5% glutaraldehyde (Grade I) and 4% freshly prepared formaldehyde in 0.1 M cacodylate buffer, pH 7.2. Samples were washed in cacodylate buffer, dehydrated in an ethanol series and embedded in a Polybed 812 resin. Thin sections were stained with lead citrate and uranyl acetate followed by observation in a Zeiss EM 900 transmission electron microscope, operating at 80 kV.

### RHBP and vitellogenin/vitellin quantifications

10 μl of hemolymph of silenced and control females were collected 7 days after the blood meal and diluted in 250 μl TBS (Tris-HCl 10 Mm, NaCl 150 mM, pH 7.4) containing approximately 8 mg of phenylthiourea. The samples were centrifuged 2x at 13.800 x g for 5 minutes at 4°C and 200 μl of the supernatants were loaded in the column for analysis as described below. The secreted protein samples from whole mount fat bodies were obtained as previously described by [36]. Briefly, dissected fat bodies from 3 days after the blood meal (day where the secretion of yolk proteins is at its maximum) were incubated in culture medium for 2 h at 28°C (5 organs for 250 μl of medium). The secreted proteins in the culture medium (200 μl) were loaded in the column and analyzed as described below. For the oocytes, 5 chorionated oocytes were homogenized in 250 μl of TBS. After centrifugation, 200 μl of the supernatant were loaded in the column. All samples (hemolymphs, fat bodies and chorionated oocytes) were loaded on a Superdex 75 10/300GL (GE HealthCare) column and analyzed by HPLC using a LC-10AT device (Shimadzu). For RHBP, the area of its 412 nm-absorbing Soret peak was used for relative quantifications. For vitellogenin, its characteristic peak, as compared to the profile of a standard purified vitellogenin detected at 280 nm, was used for the quantifications.

### Fluorescein isothiocyanate (FITC) *in vivo* oocyte uptake

FITC stock solution was prepared in DMSO at 0.5 μg/ ml. 4 μl of this solution (2 μg) were injected in the hemocoel of silenced and control vitellogenic females 8 days after the blood meal. The ovaries were dissected 18 h after the injection and observed under the fluorescence stereomicroscope (LEICA M165 FC).

### Statistics

The relative expression and ΔC_t_ values were calculated from obtained C_t_ (cycle threshold) values. The C_t_ mean values obtained from the experiments were compared using One-way ANOVA followed by Tukey's multiple comparison test. Differences were considered significant at *P*< 0.05. The relative expression values (2^-ΔCt^) were used only for graph construction. Other results were analyzed by Student’s t-Test for the comparison of two different conditions and One-way ANOVA followed by Tukey's test for the comparison among more than two conditions. Differences were considered significant at *p*<0.05. All statistical analyses were performed using the Prism 5.0 software (GraphPad Software).

## Results

### RpATG6 is highly expressed in the ovary

**Fig 1:**
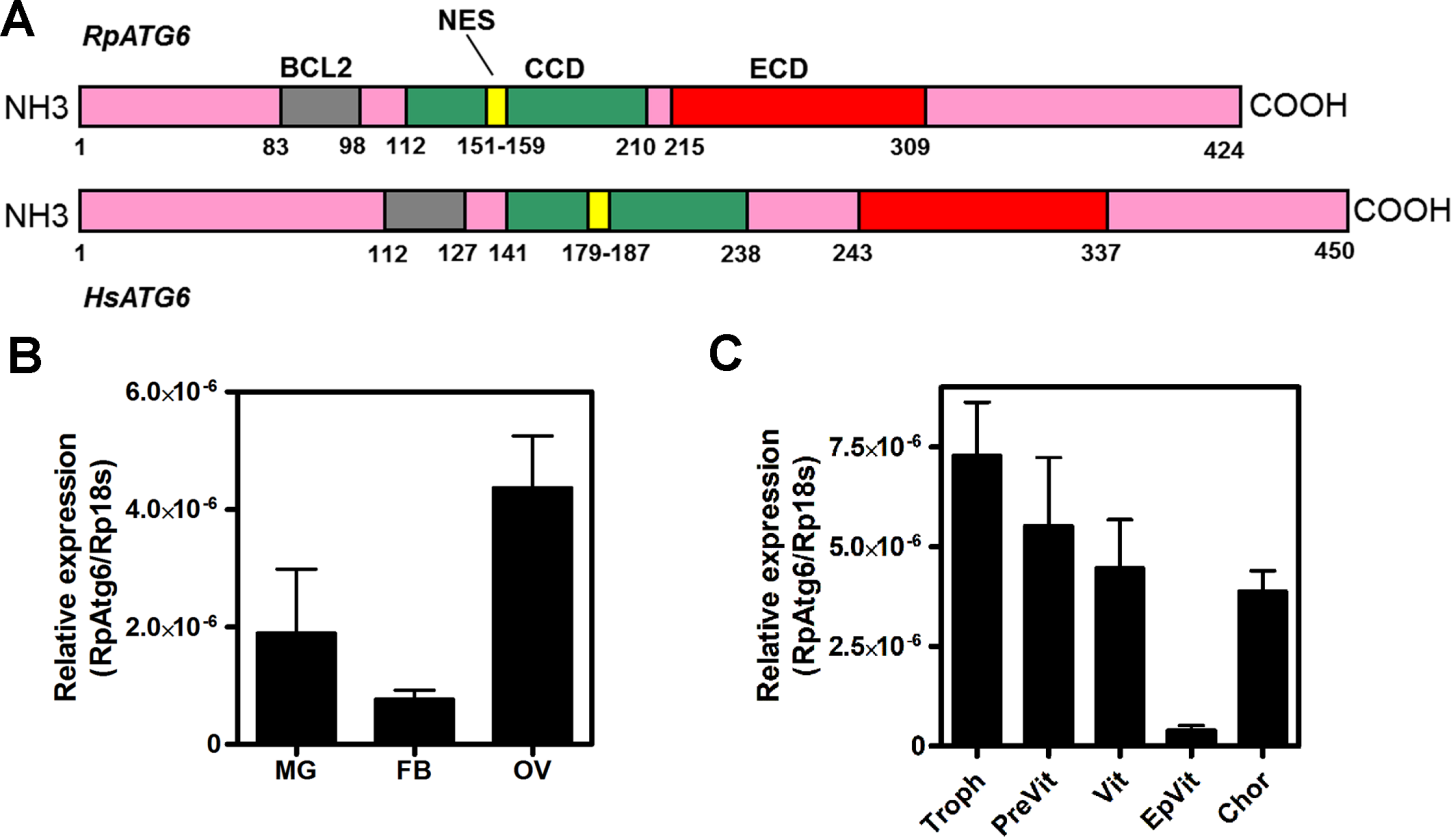
RpATG6 is highly expressed in the ovary of *Rhodnius prolixus*. **A.** Schematic diagram of the predicted conserved functional domains of RpAtg6 and HsAtg6/Beclin1 (Gene ID: 8678). BH3, BCL-2 homologous domain; CCD, coiled-coil domain; ECD, evolutionarily conserved domain. **B.** qPCR showing the relative expression of RpATG6 in different organs. MG, Midgut; FB, Fat body; Ov, Ovary. **C.** qPCR showing the relative expression of RpATG6 throughout oogenesis. Troph, tropharium; PreVit, pre-vitellogenic oocytes; Vit, vitellogenic oocytes; EpVit, follicular epithelium of the vitellogenic oocyte; Chor, chorionated oocytes. The relative expression was quantified using the ΔCT method with Rp18S as endogenous control. Graphs show mean ± SEM (n = 3).

We first identified the sequence of RpATG6 from the *R*. *prolixus* digestive tract transcriptome database [37] (GAHY01001036.1). We found one isoform of the gene ATG6 in the *Rhodnius* genome assembly (Rpro C3), with a total of 9 exons in the scaffolds KQ0344093 (exons 1–7) and ACPB3032647 (exons 8–9). RpATG6 predicted protein has 80%/ 70% similarity/ identity with the human ATG6 (Beclin1) (S1 Fig), where all the expected Atg6 conserved domains (BCL2, NES, CCD and ECD, Pfam: PF04111) were detected (Fig 1A). Quantitative PCR showed that the ovary of *R*. *prolixus* expresses an average of 2x and 5x more RpATG6 than the midgut and fat body, the other two major organs of the adult insect (Fig 1B). Throughout oogenesis, RpATG6 mRNA was detected in the tropharium (structure where the germ cell cluster and the nurse cells are located) and in all stages of the developing oocytes (pre-vitellogenic, vitellogenic and chorionated) (Fig 1C). In this experiment, we dissected the ovarioles and separated the follicles (oocyte plus follicular epithelium) to extract the total RNA. To investigate if the detected mRNA was from the oocyte or from the follicular epithelium, we dissected the follicular epithelium alone, and performed qPCRs in this tissue separately. Interestingly, we found that it accumulates only 10% of the RpATG6 mRNA detected in the whole follicle (Vit), showing that most of the mRNA detected is present in the oocyte (Fig 1C).

### Silencing of RpATG6 results in abnormal oocytes and accumulation of the main yolk proteins in the vitellogenic hemolymph

**Fig 2:**
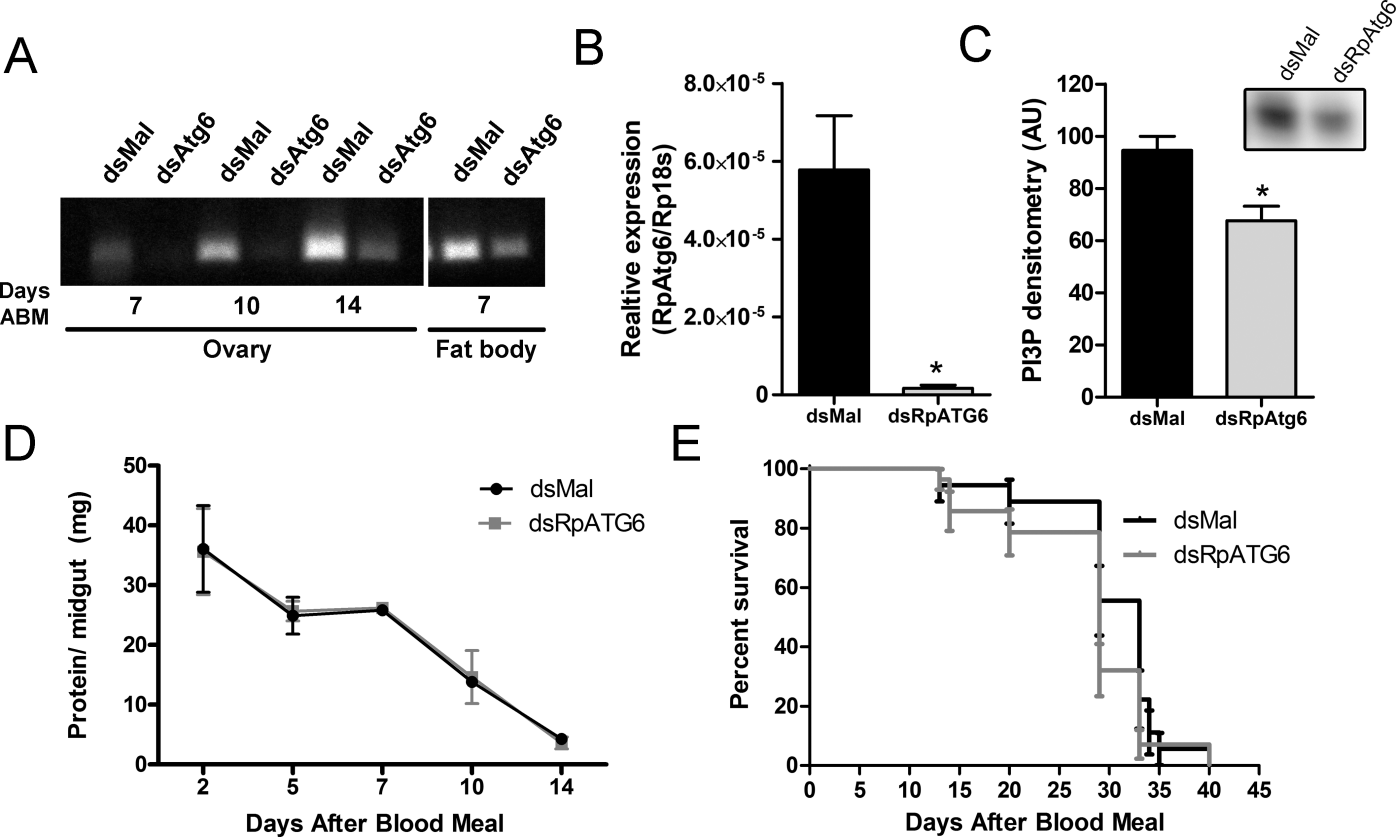
RNAi knockdown of RpATG6 does not affect major physiological processes of *R*. *prolixus*. **A.** PCR showing the knockdown efficiency of RpATG6 in the ovary and fat body at different days after the blood meal. **B.** qPCR showing the relative expression of RpATG6 in the ovary (7 days after the blood meal). **C.** PI3P detection by TLC and densitometric quantification (n = 2). **D.** Effect of RpATG6 knockdown in the blood protein digestion in the midgut. Graph shows mean ± SEM (n = 3). **E.** Survival curve of silenced females (n = 3). *p<0.05, t-Tests.

To investigate the role of RpATG6 during oogenesis, we synthesized a specific double-stranded RNA designed to specifically target the sequence of RpATG6 and injected it directly to the females haemocoel two days before the blood meal. To check the efficiency of the RpATG6 knockout we dissected the ovary and fat body (the two organs directly related to the yolk synthesis and accumulation) at different days after dsRNA injection. RpATG6 was 98% silenced in the ovary at day 7 after the blood meal (Fig 2B), and its expression levels were partially recovered at day 14 (Fig 2A). On the other hand, at day 7, RpATG6 was only moderately silenced in the fat body (Fig 2A). Accordingly, PI3P, the product of PI3K complexes (where RpATG6 is functional), was detected by TLC and shown to be 28% decreased in silenced ovaries (Fig 2C). We did not detect any difference in the main physiological characteristics of *R*. *prolixus* such as protein blood digestion and longevity (median survival of 33 days for control females and 29 days for silenced females, p>0.05), when compared to control animals (Fig 2D and 2E, respectively).

**Fig 3:**
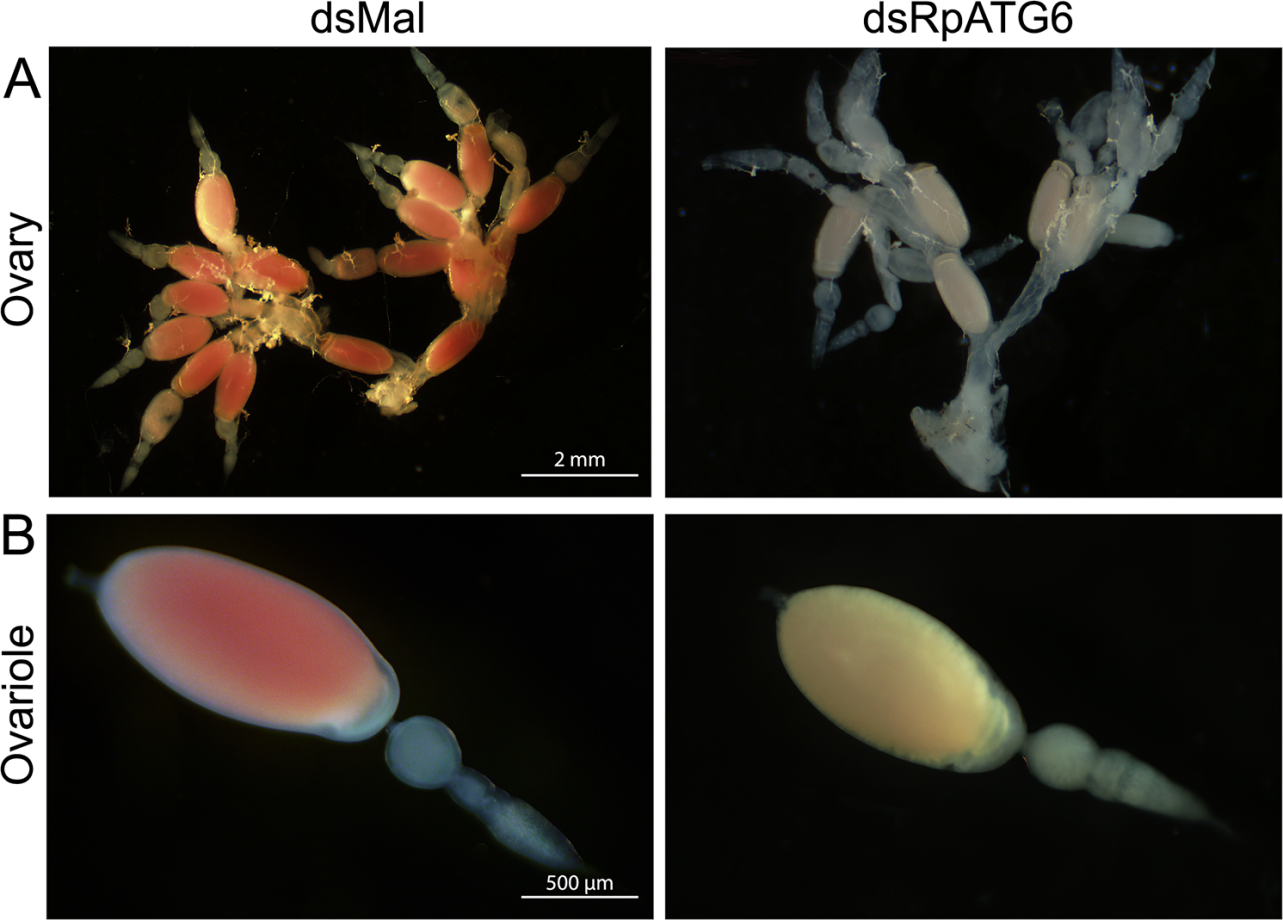
Silencing of RpATG6 resulted in abnormal morphology of the oocytes. **A.** Ovary of females previously injected with dsMal or dsRpATG6 dissected 7 days after the blood meal. **B.** Detail of the ovariole of females previously injected with dsMaL or dsRpATG6, 7 days after the blood meal.

Because day 7 after blood meal was the day where gene silencing was more effective, we dissected the silenced females at this time point and found that their oocytes were smaller and white, contrasting to the standard pink oocytes from control animals (Fig 3). The absence of the red pigment indicates the lack of one of the main yolk proteins, named RHBP (*Rhodnius* heme-binding protein). RHBP is known for being the one red molecule from the oocytes that go through the classic vitellogenesis route: they are synthesized by the fat body, secreted to the hemolymph and endocytosed by the oocytes during oogenesis [38–40].

**Fig 4:**
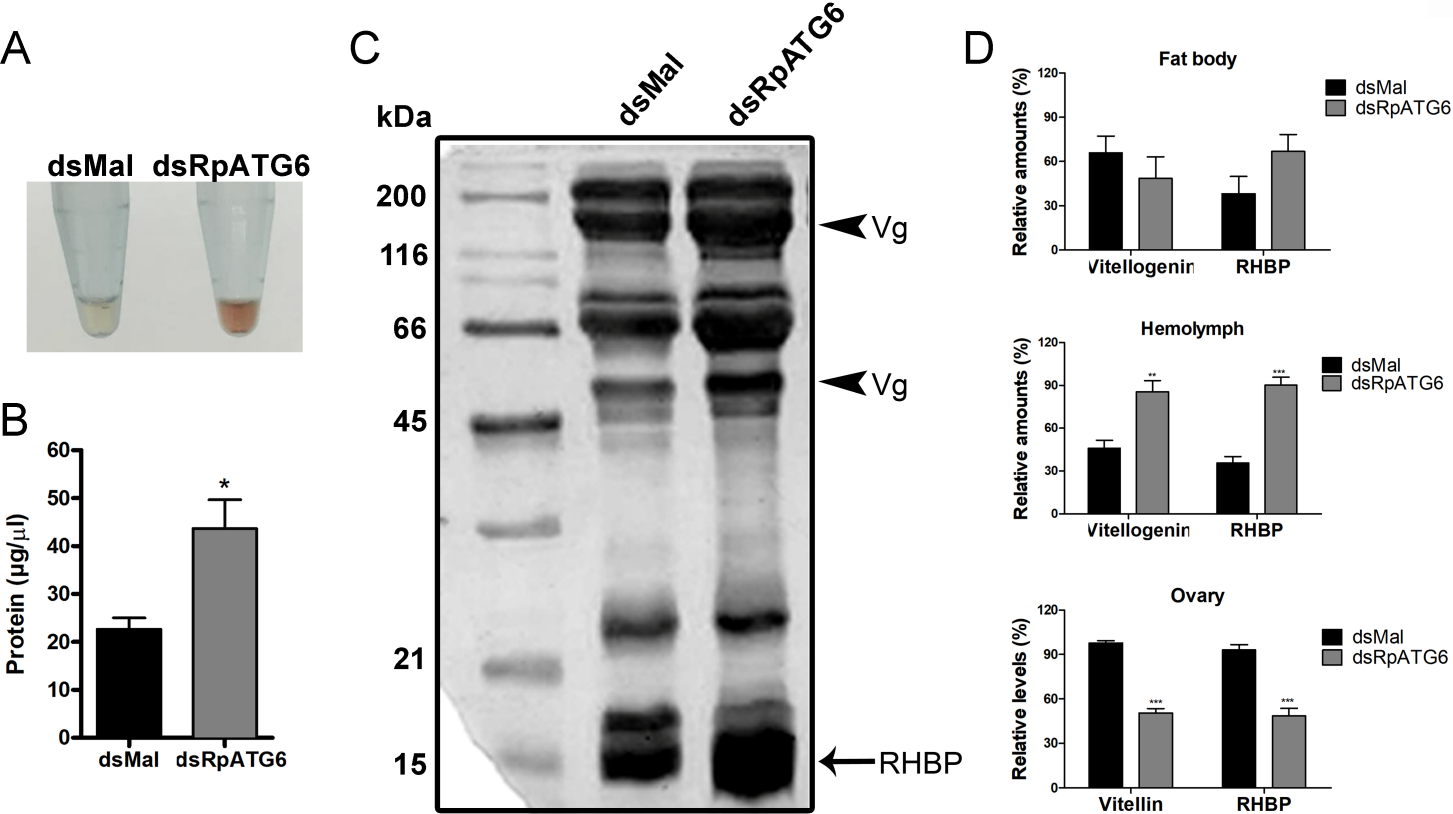
Silencing of RpATG6 leads to accumulation of yolk proteins the hemolymph. **A.** Representative images of hemolymphs extracted from females previously injected with dsMal or dsRpATG6. **B.** Total amount of protein in the hemolymph from silenced and control females. Graph shows mean ± SEM (n = 3). *p<0.05, t-Test. **C.** 13% SDS-PAGE showing the protein profile of the hemolymph from silenced and control females. All experiments were done at 7 days after the blood meal. VG, vitellogenin subunits; RHPB, Rhodnius heme binding protein. **D.** RHBP and vitellogenin/vitellin quantifications in the fat bodies (secreted samples), hemolymphs and chorionated oocytes from silenced and control females. Graphs show mean ± SEM (n = 3). *p<0.05, **p<0.01, ***p<0.001, t-Test.

We asked if the absence of RHBP in the oocytes was the result of poor uptake of macromolecules from the hemolymph or a defect in the synthesis of RHBP by the fat body. Thus, we looked at the total protein profile in the hemolymph of silenced vitellogenic females 7 days after the blood meal. High concentrations of RHBP were already apparent in the freshly extracted hemolymph from silenced females, as they were pink (Fig 4A), and had roughly twice the protein concentration of the hemolymph from control animals (Fig 4B). SDS-PAGE of the hemolymph shows that silenced females accumulated the major secreted yolk proteins—RHBP (arrow) and vitellogenin (two subunits, arrowheads) (Fig 4C). Quantifications of RHBP and vitellogenin/ vitellin in the hemolymph showed that silenced females accumulate approximately twice the levels of both proteins, when compared to control females (Fig 4D, upper panel). Accordingly, chorionated oocytes store only half the amount of the two major yolk proteins (Fig 4D, middle panel). To test the levels of the secreted yolk proteins by the fat bodies, the dissected organs were incubated for 2 h in culture medium (using a protocol previously described by [36]) and the secreted protein profiles were analyzed by HPLC. We found that the levels of both secreted vitellogenin and RHBP by the fat bodies were similar between silenced and control females (Fig 4D, lower panel).

### RpATG6 silenced oocytes are not able to accumulate the main yolk macromolecules and are not viable

**Fig 5:**
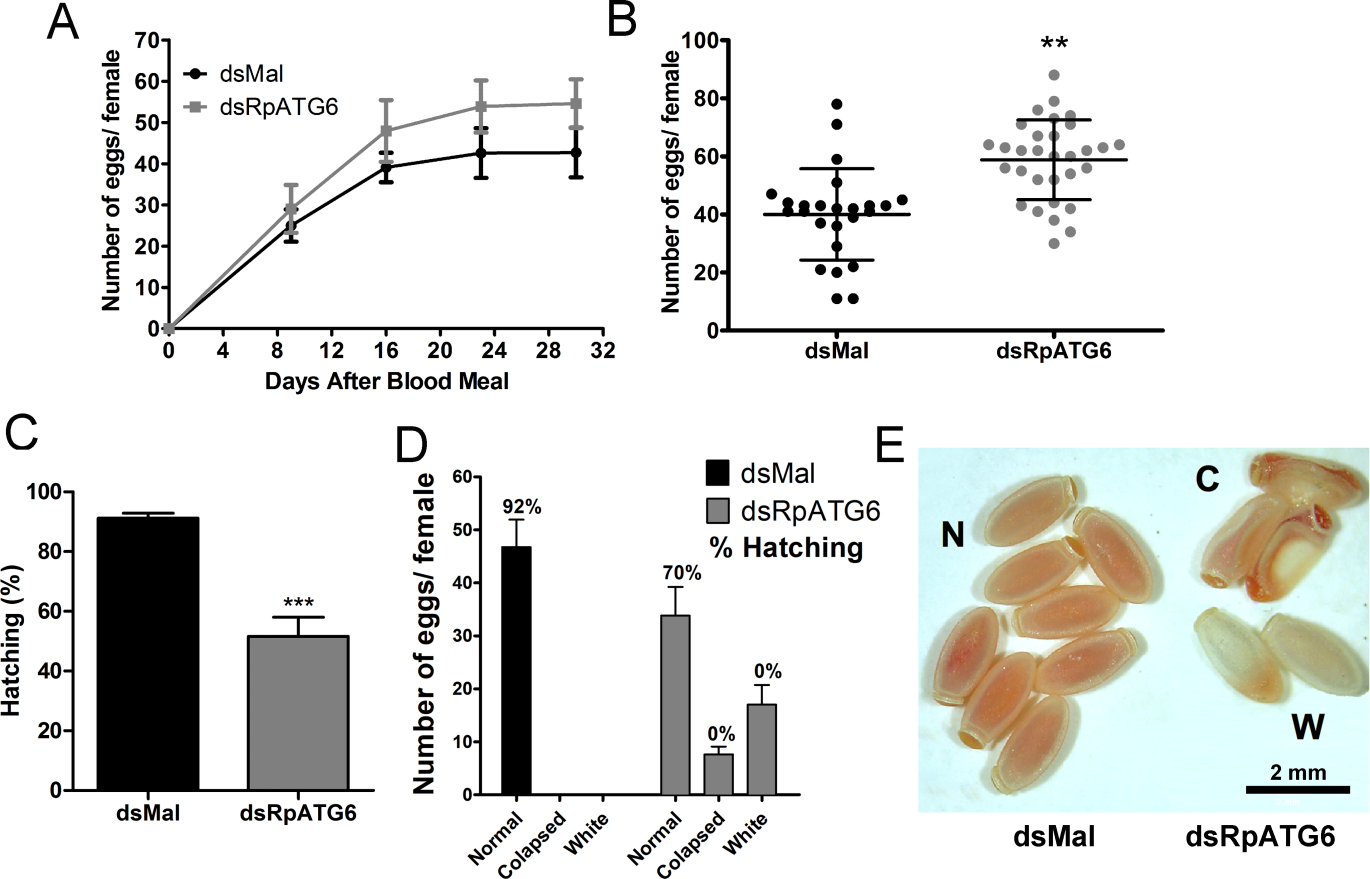
Silencing of RpATG6 increases oviposition but decreases embryo viability. The oviposition and hatching were monitored after the blood meal. **A.** Number of eggs laid per female over 4 weeks. Graph shows mean ± SEM (n = 4). **B.** Total of eggs laid by silenced and control females. Graph shows mean ± SEM (n = 25). **p<0.01, t-Test. **C.** Hatching rates after silencing of RpATG6. Graph shows mean ± SEM (n = 3). ***p<0.001, t-Test. **D.** Phenotypic distribution observed after knockdown of RpATG6. Percentage (%) of hatching per phenotype is also showed. **E.** Representative image of the phenotypes observed in the eggs. (N), normal (control) eggs; (C), collapsed eggs, (W), white eggs.

To investigate the role of RpATG6 on oviposition and hatching, we quantified the eggs laid individually by females and maintained the collected eggs under ideal conditions to ensure embryo development. Surprisingly, our results show a 28% increase in the number of eggs laid by silenced females (Fig 5A and 5B). Despite the higher oviposition, silenced females transiently laid eggs presenting two main types of abnormal morphology: an average of 18 oocytes (31% of the total number of eggs) where white and 8 (13% of the total number of eggs) where collapsed (Fig 5D and 5E). In total, we observed a decrease in 50% in embryo viability (Fig 5C), but all eggs that presented one of the phenotypes described above (white or collapsed) are unviable (Fig 5D, see numbers on the top of the bars for the hatching % for each of the phenotypes).

**Fig 6:**
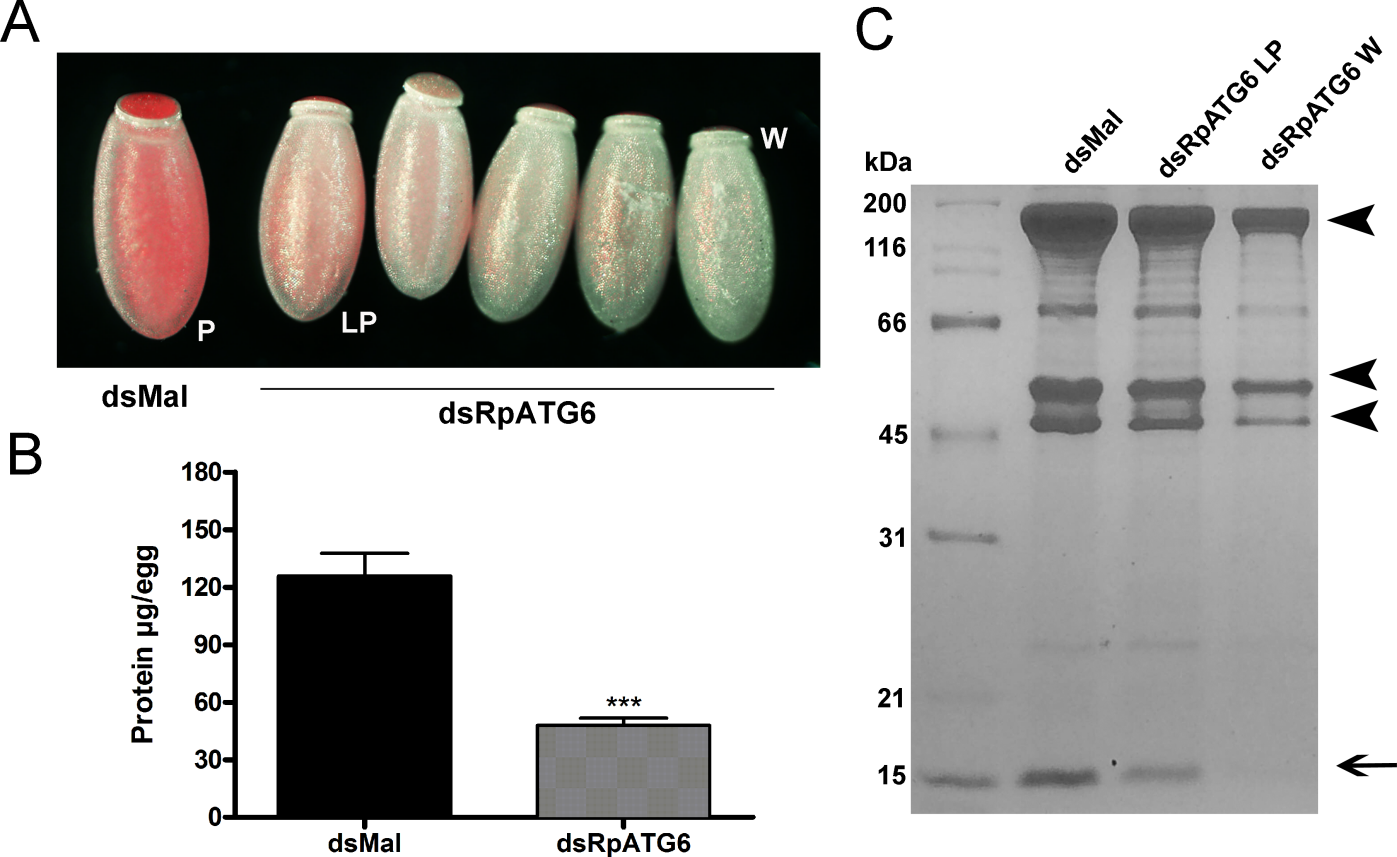
Silencing of RpATG6 affects the yolk protein content of the eggs. **A.** Representative images of the gradual phenotype observed in the eggs of females previously injected with dsRpATG6. P, pink egg (control); LP, light pink egg; W, white egg. **B.** Total amount of protein in the white eggs of females previously injected with dsMal or dsRpATG6 measured by the Lowry (Folin) method. Graph shows mean ± SEM (n = 3). ***p<0.001, t-Test. **C.** 13% SDS-PAGE showing the protein profile of 24h eggs from females injected with dsRNAs. dsMal (control pink eggs), dsRpATG6 light pink eggs (dsRpATG6LP) and dsRpATG6 white eggs (dsRpATG6W). Arrowheads, Vitellin subunits. Arrow, RHPB–Rhodnius Heme Binding Protein.

RpATG6 RNAi silencing triggers a gradual phenotype that can be easily seen in the varying color of the laid eggs (conveniently, because RHBP is red). Starting at day 7 after blood meal, silenced females started to lay eggs with a lighter pink color. In the next 5–6 days, the eggs were laid with a progressive lighter pink color; up to the day when they were laid completely white (Fig 6A).

White eggs accumulated only 35% of the total protein found in the control eggs (Fig 6B), and the accumulation of the main yolk proteins, vitellin (arrowheads) and RHBP (arrow), was severely compromised (Fig 6C). We also predicted the volume of the silenced eggs and found that the white eggs are 38% smaller, with an average volume of 1.03 ± 0.06 mm^3^ versus 1.66 ± 0.09 mm^3^ of the eggs laid by control females.

**Fig 7:**
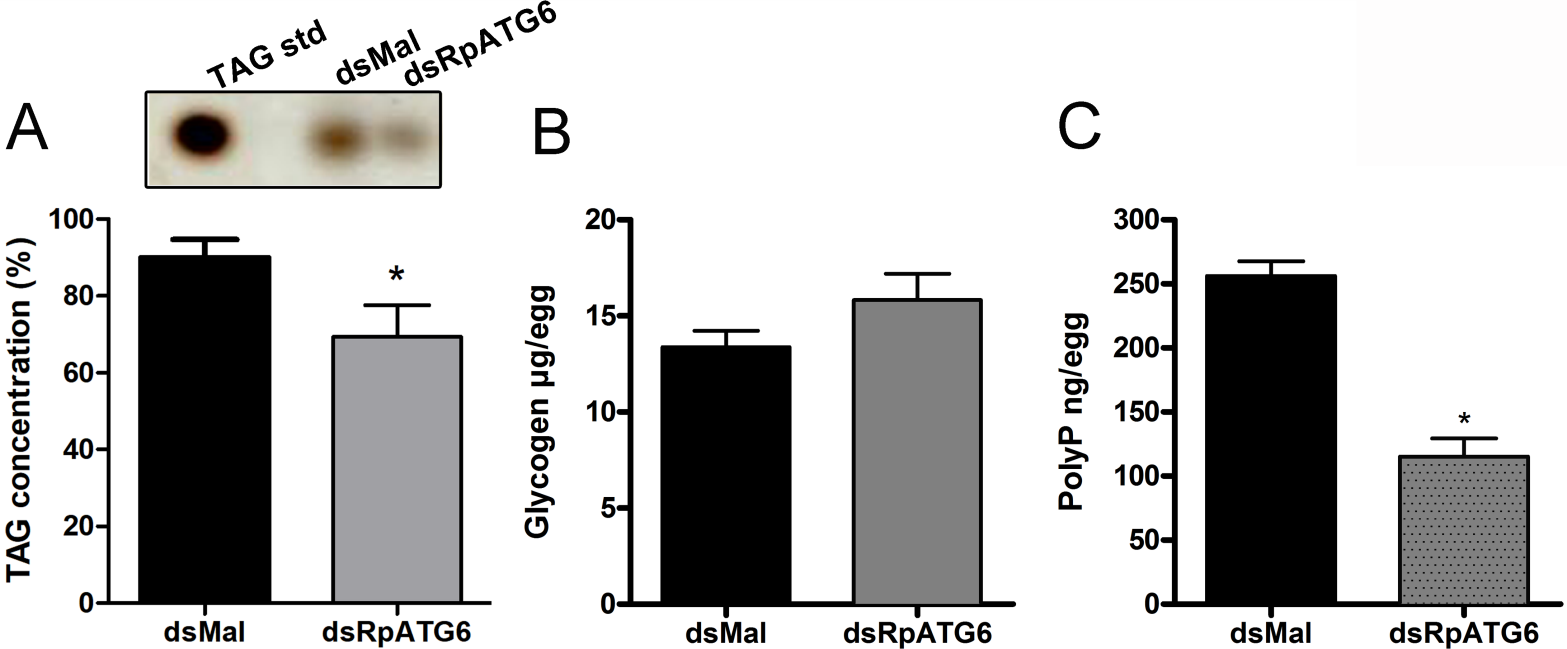
Knockdown of RpATG6 leads to a decrease in the accumulation of TAG and PolyP but not in the glycogen content of the eggs. **A.** Densitometry measurements of the TAG content in the silenced eggs after neutral lipid separation by TLC. Upper panel: inset of representative TAG spots in the TLC. **B.** Glycogen content of the eggs of females previously injected with dsMal or dsATG6. **C.** Total PolyP content of the eggs of females previously injected with dsMal or dsATG6. All graphs show mean ± SEM (n = 3). *p<0.05, t-Tests.

In addition to the yolk proteins, carbohydrates and lipids are also an important part of the yolk storage. To test the effect of RpATG6 in the buildup of non-protein yolk macromolecules we measured the content of TAG and glycogen in the white eggs. We found no differences in the glycogen accumulated in white silenced eggs (Fig 7B) and a 20% reduction in the TAG reserves in white eggs (Fig 7A). Another major yolk component is the polymer PolyP, which is known to provide indispensable phosphate supply to the high metabolic demands of the embryo. Silenced eggs showed a 50% decrease in its PolyP levels when compared to control eggs (Fig 7C).

### RpATG6 is important for the biogenesis of the endocytic vesicles and formation of yolk organelles in the oocyte cortex

**Fig 8:**
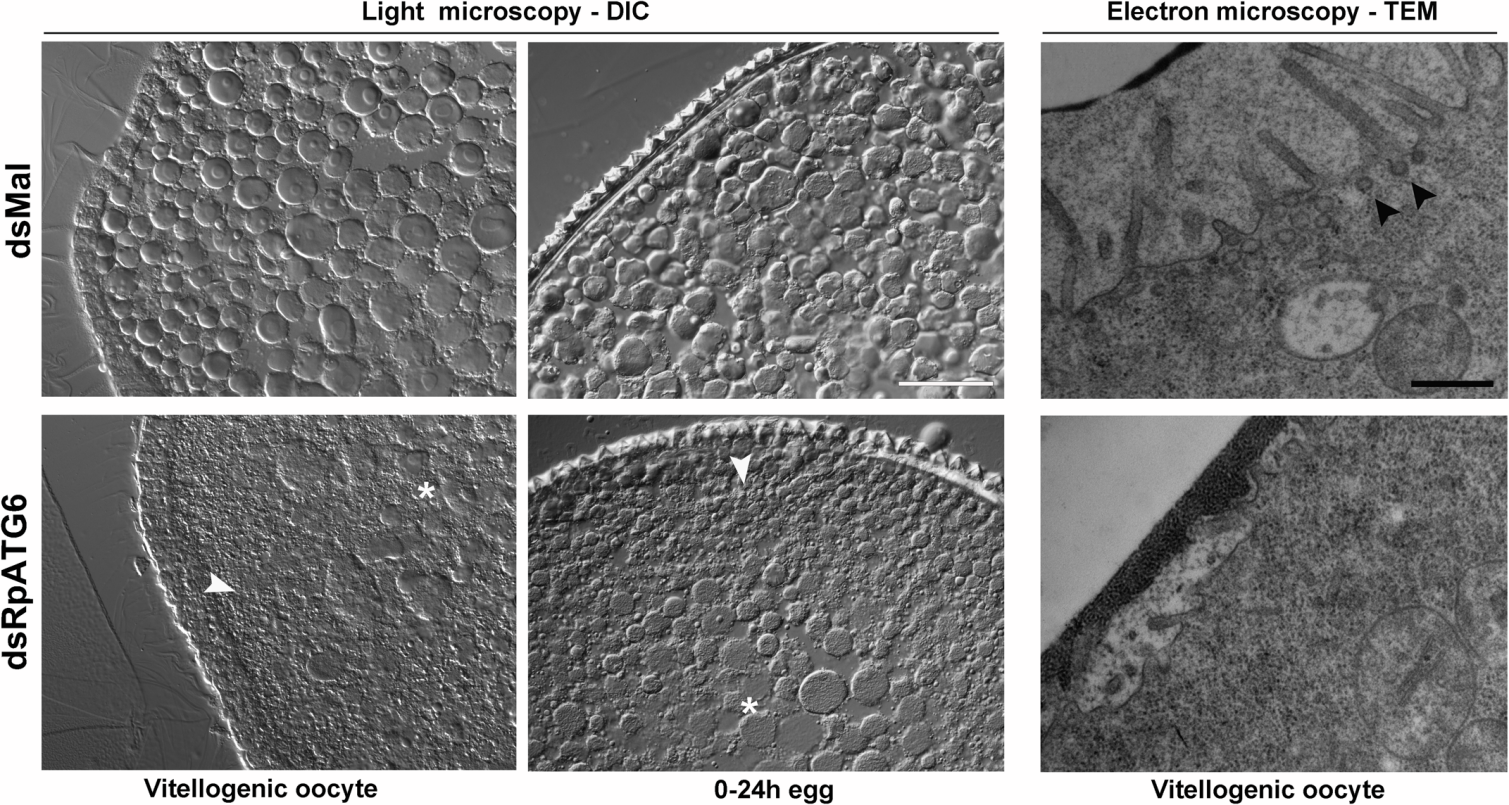
Knockdown of RpATG6 results in abnormal distribution of the yolk organelles and less endocytic vesicles in the oocytes. **Left panel:** Representative images of cross-sections of early vitellogenic oocytes and 24h-eggs from control and silenced females were observed in the light microscope. The images show an accumulation of larger yolk organelles in the core cytoplasm (*) and an irregular distribution of these organelles in the periphery of dsRpATG6 oocytes and eggs (white arrowheads). Bars: 200 μm. **Right panel:** Representative images of cross-sections of cortex and plasma membrane of vitellogenic oocytes from control and silenced females observed under the TEM. Silenced oocytes do not show projected microvilli or endocytic vesicles as seen in the control oocytes. Black arrowheads: endocytic vesicles. Bars: 0.5 μm.

**Fig 9:**
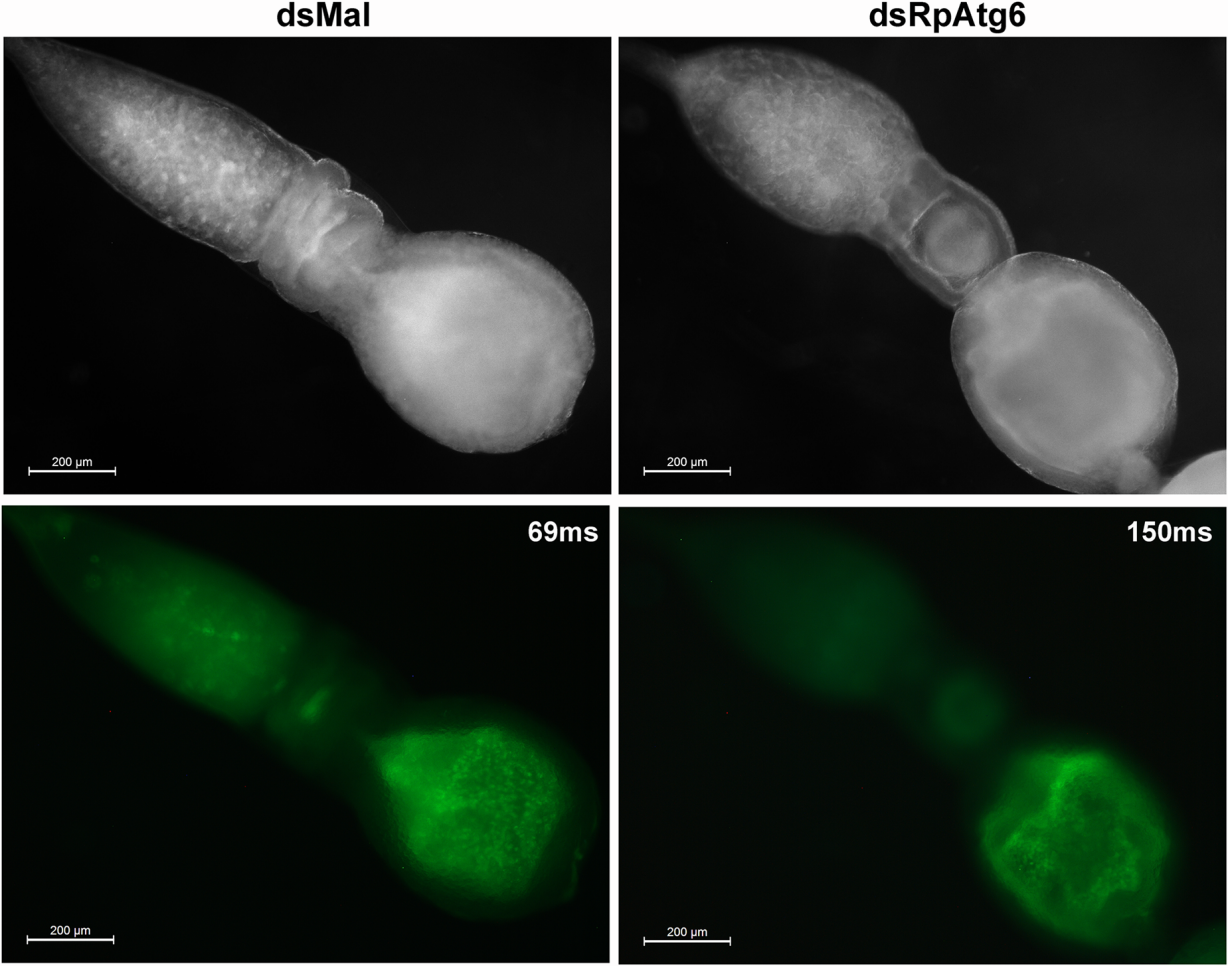
*In vivo* FITC-uptake by the oocyte yolk organelles. Silenced and vitellogenic females were injected with 2 μg of FITC and their ovaries were dissected 18 h later for observation under the stereomicroscope. Note the abnormal morphology of the endocytic-originated yolk organelles in the early vitellogenic oocytes from silenced animals (dsRpATG6) when compared to control females (dsMal). Upper panel: brightfield images. Lower panel: fluorescence images. The exposure time (in milliseconds) for the fluorescence images are indicated.

Because the lack of RpATG6 is apparently affecting the endocytosis of the yolk macromolecules by the oocytes, we decided to look at the yolk organelles in the silenced (white) vitellogenic oocytes and 24 h eggs. Transversal cross sections showed an accumulation of larger yolk organelles in the core cytoplasm and an irregular distribution of these organelles in the periphery of oocytes and eggs (Fig 8, left panel). This morphology suggests problems in the biogenesis/ sorting of the yolk organelles, compatible with the hypothesis that silenced oocytes were not able to properly recruit the endocytosis machinery during oogenesis. Vitellogenic oocytes were also processed for transmission electron microscopy. High resolution images from the plasma membrane and cortex showed that the silenced oocytes do not present the prominent microvilli and the endocytic vesicles (arrowheads) that can be found in the control oocytes (Fig 8, right panel). To further investigate the morphology of the yolk organelles in silenced oocytes we injected FITC in the vitellogenic females and dissected their ovaries 18 h later. We found that the FITC labeled yolk organelles presented, as previously observed in the cross sections, irregular morphology and distribution in the cytoplasm of oocytes from silenced females, when compared to control oocytes (Fig 9).

## Discussion

The mRNA of the one single isoform of the *RpATG6* gene in the *Rhodnius* genome assembly was detected in all major organs of the adult insect, but its expression levels are 2x and 5x higher in the ovaries of vitellogenic females than in the midgut and fat body. Accordingly, the only apparent phenotype observed (even though silencing was systemic) was in the impairment of the uptake of yolk macromolecules by the oocytes. No effects on the major physiological routes of the adult animal were apparent: blood digestion, yolk protein synthesis and longevity were not altered. Similar results were previously found in the hard tick *Haemaphysalis longicornis*, where the lack of ATG6 resulted in a decrease of internalization yolk proteins by the oocytes [18]. Atg6 is a key component of two different PI3K complexes that are essential for autophagy or endocytosis, but how the formation of the different complexes is regulated is still unclear [9, 17]. Endocytosis and the subsequent membrane endosomal traffic are integral processes to eukaryotic cells and are especially important in the context of the oocyte maturation and yolk accumulation during oogenesis. PI3P-kinases complexes are central to this process as the local production of PI3P is known to recruit specific effector proteins that promote endocytosis, endosome fusion, endosome motility and endosome maturation [41], as well as actin dynamics, which allows membrane deformations for cell protrusions and vesicle formation [42]. Because RpATG6 is part of a class III PI3P kinase complex, it was expected that its silencing would result in a decrease in the levels of PI3P in the oocytes, as we detected by TLC, and the lack of the that signal molecule is likely the reason why microvilli, endocytic vesicles and the yolk organelles in the cortex of silenced oocytes are not properly formed.

Although our data indicates impairment in the recruitment of the endocytic machinery in the oocytes, it does not exclude the possibility of deleterious effects also in autophagy. It is important to note that our experiment was set to focus on vitellogenesis, so we triggered the knockout right at the time that females were fully fed and, therefore, producing the oocytes. At this nutritional status, somatic tissues are only going through background levels of autophagy, so it is expected that the lack of RpATG6 (or any other ATG) would not result in any major autophagy-related phenotype.

RpATG6 high expression levels in the tropharium, when compared to the oocytes, is interesting in the sense that in a meroistic-telotrophic type of ovary, the accessory nurse cells are located in the tropharium being connected to the oocytes through cytoplasmic bridges. Since the oocyte itself is undergoing meiosis during oogenesis most of its accumulated mRNA is synthesized by the nurse cells, and it is possible that the high levels of RpATG6 mRNA found in the tropharium is targeted for delivery to the oocytes [43]. It is also interesting to notice that we observed a transient phenotype (probably because of silencing recovering), where the silenced females produced a batch of oocytes (from 7–12 days after feeding) that were not able to properly take up the yolk macromolecules. So, it is possible that the spare yolk accumulated in the hemolymph was used for the animal to build more eggs after the silencing effect was lost, and that this is the reason why the silenced females laid more eggs in total when compared to control females.

Apart from the main yolk proteins vitellin and RHBP, we also observed a decrease in the storage of TAG and PolyP, both major non-protein yolk components of the oocytes. 30–40% of the dry weight of the full grown oocyte is comprised by lipids, mostly fatty acid, cholesterol, phospholipids and triacylglycerol (TAG) [44]. Among those lipids, TAG is the main yolk lipid and one of the major sources of energy to the embryo. PolyP is a polymer of phosphate residues linked by high-energy phosphoanydride bonds, which has been found in a wide diversity of organisms [45]. Amongst several other functions, PolyP has been described as one of the yolk storage macromolecules in insects, as its pools are degraded during early embryogenesis as a source of Pi to the embryo anabolic metabolism [46]. Because it is known that not all yolk macromolecules are delivered to the oocytes through an endocytic pathway, our findings suggest that: 1) the silencing of RpATG6 may impair a different mechanism that regulates the delivery of TAG and PolyP to the oocytes; or 2) TAG and PolyP may be carried into the oocyte by a macromolecule that is being internalized through endocytosis. The yolk vitellin is the internalized post-translationally altered form of the circulating vitellogenin. It is know that TAG and phospholipids are bound to the internalized vitellogenin [47], and that large amounts of vitellogenin are taken up by insect oocytes. Thus, it is tempting to hypothesize that the decrease in the vitellin pools in the silenced oocyte could be at least partially responsible for the reduction in the TAG storage in the silenced eggs. However, because of its low lipid content, it is known that insects vitellogenins contribute only to a small amount of the lipids in the egg storage [44]. In this context, the possibility of the internalization of lipophorin (Lp), the main insect lipoprotein, by endocytosis via Lp Receptor (RLp), should be taken into account. Previous works on *R*. *prolixus* excluded this process based on the evidence that 1) injected [^32^P] Lp was not accumulated inside the oocytes [48] and 2) immunogold electron microscopy labeled only the surface of the oocytes [49]. However, recent data found evidence that endocytosis of Lp by oocytes seems to occur in most insect species [44]. Also, a Lp receptor was recently found in the genome of *R*. *prolixus* (RLp Gene: LpR1 RPRC011390, Vector Base). Thus, we cannot rule out the possibility that maybe a small fraction of the lipids in the oocytes of *R*. *prolixus* is delivered through Lp endocytosis.

As for PolyP, it is possible that it is also linked to vitellin, but it has never been properly tested. Further experiments testing the synthesis and delivery mechanism of PolyP to the oocytes are necessary to understand their fate in the oocytes. Glycogen also comprises the yolk, but while extra ovarian organs produce proteins and lipids, glycogen is synthesized in the ovary itself [50]. The accumulation of carbohydrates by the oocytes is still not clear but it is known that a glycoside hydrolase activity is required to the transport of sugar during oogenesis in *R*. *prolixus* [51]. Accordingly, we found that the levels of glycogen in the silenced oocytes remain unaltered, gathering evidence that the delivery of carbohydrates to the oocytes is independent from an endocytic pathway.

Our findings that the microvilli and endocytic vesicles are not properly formed in the silenced oocytes, and that part of the yolk organelles in the oocyte cortex have abnormal morphology further support the assumption that endocytosis was compromised. It is generally acknowledged that the mature oocyte comprises different types of what we collectively call yolk organelles, and that this is the result of distinct packaging routes during oogenesis where the size and localization of the yolk vesicles within the oocyte is maternally-determined. Several groups have described that the yolk organelles population is not homogeneous in the fertilized egg. The vesicles can vary in its macromolecule contents, density and size, and for several models it is possible to fractionate the yolk organelles in accordance with their difference of size and density [52–56]. In the stick insect, *Carausius morosus*, differential acidification of the yolk organelles was already described and correlated with proteolytic activity [57]. In *Periplaneta americana* and *R*. *prolixus*, some small yolk organelles were described as acidocalcisome-like organelles, and they can be fractioned by differential centrifugation [58, 59]. For the hard tick *Boophilus microplus* the proteolytic activity has also been correlated with differential acidification of yolk granules and with vesicles in the cortex of the fertilized egg [60]. At early development, the periphery of the egg is the region where the blastoderm is formed, so it makes sense that cortex organelles would be the ones acidifying during development to trigger the yolk mobilization. Because the silencing of RpATG6 apparently leads to defects in the formation of primarily a set of yolk organelles (the ones in the cortex), it is possible that RpATG6 may be involved in the biogenesis of the different populations of yolk organelles.

Altogether, we found that RpATG6 has a key role in the uptake of the main yolk proteins and in the biogenesis of the yolk organelles in *R*. *prolixus*. Since the yolk storage and degradation are crucial events for proper oocyte formation and embryo development, understanding the role of RpATG6 could contribute to the elaboration of vector control strategies. This is especially important in the context of Chagas Disease, in which, to this day, the main forms of control and prevention in endemic areas are vector-avoiding tactics, as the use of insecticide sprays and bednets (www.who.int/chagas/en/). Such approaches are virtually the same ones that have been done in the past decades, so it becomes evident how little accomplishments we have made in our efforts to manage this insect population and dispersion. Certainly, that lack of progress is at least partially due to our scattered knowledge regarding the insect reproduction biology from a molecular point of view. In this context, we think that contributions to this field are of key importance and may help us to further understand vectors biology and to elaborate new tactics for population control and the prevention of vector-borne NTDs such as Chagas Disease.

## Supporting information

S1 Fig RpATG6 sequence and alignments with ATG6 orthologues from different species. **A.** Multiple sequence alignment of conserved domains Coiled Coil Domains (CCD), Nuclear Export Signal (NES), Becl2-homology-3 motif (BH3) and Evolutionarily Conserved Domain (ECD) in *Rhodnius prolixus*, *Drosophila melanogaster* and *Homo sapiens*. The structure of the different domains of ATG6/Beclin1 was previously described in *Homo sapiens* [8] (*) corresponding the amino acids of the conserved domains. **B.** Matrix of similarity and identity of Atg6/Beclin1 protein sequence in different species (SIAS Server). Reference sequence *Rp–Rhodnius prolixus*. Dm–*Drosophila melanogaster*; Hs–*Homo sapiens*; Mm–*Mus musculus*; At–*Arabidopsis thaliana*; Sc—*Saccharomyces cerevisiae;* Bm*–Bombyx mori;* Am*–Apis mellifera*.
(TIF)
