# Supplementary figures and images for: Silencing of RpATG6 impaired the yolk accumulation and the biogenesis of the yolk organelles in the insect vector R. prolixus
Source: PLoS Negl Trop Dis. 2018 May 16;12(5):e0006507. doi: 10.1371/journal.pntd.0006507 (PMC5973624; doi:10.1371/journal.pntd.0006507)

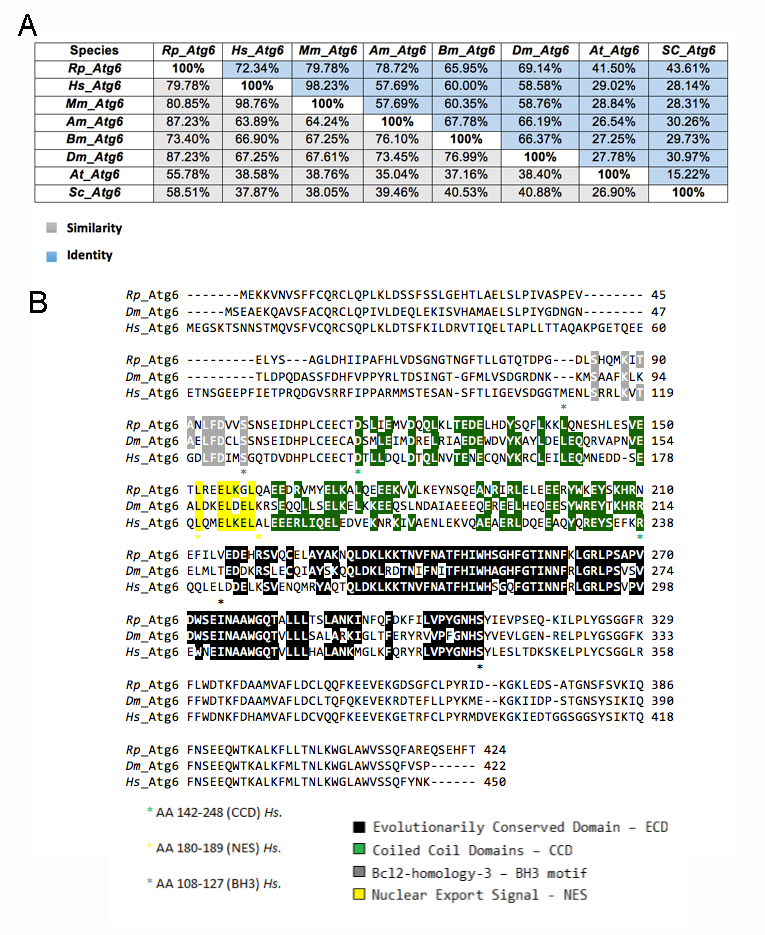

Supplement: S1 Fig — A. Multiple sequence alignment of conserved domains Coiled Coil Domains (CCD), Nuclear Export Signal (NES), Becl2-homology-3 motif (BH3) and Evolutionarily Conserved Domain (ECD) in Rhodnius prolixus, Drosophila melanogaster and Homo sapiens. The structure of the different domains of ATG6/Beclin1 was previously described in Homo sapiens [8] (*) corresponding the amino acids of the conserved domains. B. Matrix of similarity and identity of Atg6/Beclin1 protein sequence in different species (SIAS Server). Reference sequence Rp–Rhodnius prolixus. Dm–Drosophila melanogaster; Hs–Homo sapiens; Mm–Mus musculus; At–Arabidopsis thaliana; Sc—Saccharomyces cerevisiae; Bm–Bombyx mori; Am–Apis mellifera. (TIF) [file pntd.0006507.s001.tif]
